# A Multi Camera and Multi Laser Calibration Method for 3D Reconstruction of Revolution Parts

**DOI:** 10.3390/s21030765

**Published:** 2021-01-24

**Authors:** Hugo Álvarez, Marcos Alonso, Jairo R. Sánchez, Alberto Izaguirre

**Affiliations:** 1 Vicomtech Foundation, Basque Research and Technology Alliance (BRTA), Parque Científico y Tecnológico de Gipuzkoa, Paseo Mikeletegi 57, 20009 San Sebastián, Spain; jrsanchez@vicomtech.org; 2 Robotics and Automation Group, ECS Department, Mondragon University, Loramendi Kalea 4, 20500 Mondragon, Spain; malonson@mondragon.edu (M.A.); aizagirre@mondragon.edu (A.I.); 3 Computational Intelligence Group, CCIA Department, UPV/EHU, Paseo Manuel de Lardizabal 1, 20018 San Sebastian, Spain

**Keywords:** optical sensor, laser sensor, calibration, 3D reconstruction

## Abstract

This paper describes a method for calibrating multi camera and multi laser 3D triangulation systems, particularly for those using Scheimpflug adapters. Under this configuration, the focus plane of the camera is located at the laser plane, making it difficult to use traditional calibration methods, such as chessboard pattern-based strategies. Our method uses a conical calibration object whose intersections with the laser planes generate stepped line patterns that can be used to calculate the camera-laser homographies. The calibration object has been designed to calibrate scanners for revolving surfaces, but it can be easily extended to linear setups. The experiments carried out show that the proposed system has a precision of 0.1 mm.

## 1. Introduction

In the context of laser triangulation systems, the calibration of the different optical elements is critical for the accuracy of the obtained reconstructions. These systems are composed by a laser line that forms a plane that intersects with the object being measured, and a camera that captures the produced laser line interception. The calibration of these elements involves the characterization of the camera parameters and the relationship between image and laser planes.

Standard calibration methods, usually referred as “sheet of light calibration”, are based mainly on two methods. The former uses planar objects with chessboard or circular patterns that are used to calculate all the calibration parameters simultaneously [1,2,3,4,5]. Detecting these control points in the image allows obtaining the camera parameters and the pose of the calibration object with respect to the camera. At the same time, the detection of the projection of the laser plane in the calibration pattern enables us to estimate its pose with respect to the camera, completely characterizing the triangulation system. The latter employs a moving part whose geometry (usually a diamond shaped object) is well known [6]. Nevertheless, the motion of the part has to be well controlled, e.g., by means of an accurate stepper motor.

Besides that, the depth of field of standard cameras is centered on a world plane that is parallel to the image sensor. However, laser camera systems should have the depth of field focused on the laser plane. For these cases, the lens is usually tilted with respect to the image plane through the use of an adapter (or analogously, the image sensor is tilted with respect to the lens) to ensure that the desired world plane is correctly focused (Scheimpflug principle [5]). It is noteworthy that, although some authors (e.g., [2,4]) have applied standard calibration methods based on planar objects in scenarios with Scheimpflug, it is not an easy procedure. The main difficulty is to capture images of the planar calibration pattern in which a large area of the pattern is focused, which implies that the calibration plate should be placed almost parallel to the laser plane. Furthermore, there are situations where several lasers or cameras are needed because of the geometry of the object being captured. This configuration increases the complexity of the calibration since all laser and image planes must be related to each other under the same reference system. This fact implies that the control points of the calibration pattern have to be visible at the same time by the cameras tied to the same laser. However, this may not be possible due to occlusions derived from the convergence angles of the cameras with respect to the laser plane.

**Figure 1:**
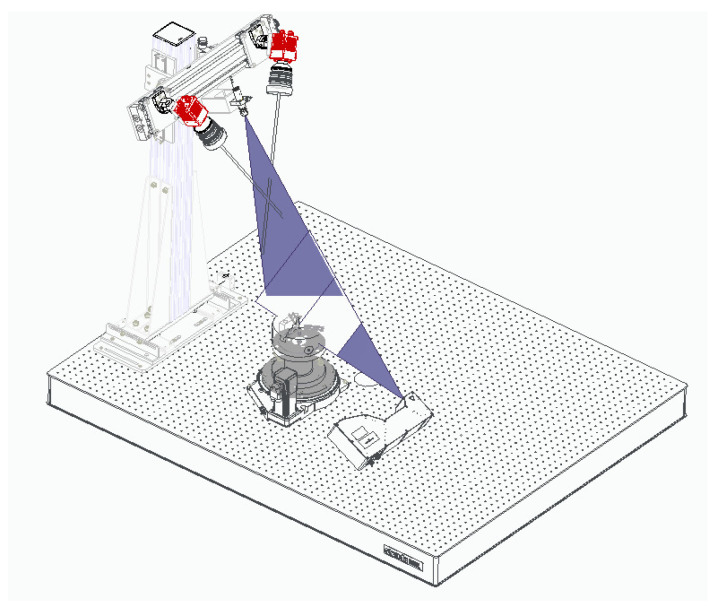
The proposed laser 3D triangulation system with 3 camera-laser pairs plus a motorized rotation stage. Two cameras and one laser have been placed at the top (to obtain the reconstruction of the interior of the object), while the remaining camera and laser have been placed at the bottom (to reconstruct the external surface of the object). The rotation stage ensures visibility throughout 360 degrees of the object.

In this work, we address the calibration of a laser triangulation system composed by three cameras and two laser lines that are configured as shown in Figure 1. This system is used to reconstruct revolution parts that are fixed in a rotating stage.

Taking this into account, we propose a calibration method that uses a 3D calibration object with a conical shape that can be used to determine the homographies [7] between laser and image planes. In order to estimate these homographies, the camera undistortion map under Scheimpflug conditions has to be identified, or sufficient conditions have to be met in which the undistortion map calculation can be discarded.

In this paper, we present a more detailed investigation of camera-laser calibration based on homographies under Scheimpflug conditions. Section 2 provides related work in camera-laser triangulation. Section 3 explains an overview of the employed calibration methodology. Section 4 summarizes the practical calibration of the real setup. Section 5 describes the usage of the 3D reconstruction system. Section 6 provides experiments and discussion, and finally, Section 7 enumerates conclusions. Additionally, please refer to [8] to see a complementary work that provides an industrial application of the presented optical system.

## 2. Related Work

A 3D triangulation system is composed of at least one laser and one camera. However, it is common to find solutions that combine multiple lasers [9,10,11,12], lasers of different colors [13] or multiple cameras [14,15] to extend the range of visibility and avoid blind areas.

Moreover, the 3D triangulation systems are generally combined with external actuators such as linear stepper motors [10,12,13,15], turntables [9,10,15,16,17] or robotic arms [11,18] to extend the scanning line to a wider regions.

There are several solutions in the literature to calibrate a 3D triangulation system identifying the relative pose between each of the devices forming the system. Although some of them perform a self-calibration (no calibration object is required), these alternatives do not recover the scale of the scene and are not of interest to this work (see [5] for an in-depth explanation). The most common way to calibrate a 3D triangulation system is to use a calibration target. Because of its simplicity and good performance, planar targets are very popular, especially chessboards [10,12,14,16] and those with dot patterns [11,13]. In some cases planar targets are not a feasible solution (for example, in an arrangement where the planar pattern cannot be seen by all cameras simultaneously), so a 3D calibration target is used instead. These 3D calibration objects are specific to each application and can be of different shapes and sizes, such as a stepped [15] or creased [18] gauge, a 3D cube with a white mark in the middle [15], a 3D sphere [17], or a 3D cone [9], among others. Regardless of the calibration object, the overall calibration procedure is similar, cameras capture the projection of the laser lines on the calibration target at different points of views (controlled by the external actuators) to identify some control points with known 3D coordinates that allow to extract the positioning of each device in a common global coordinate system. Traditionally, this procedure implies solving equations using standard mathematical tools, but with the evolution of the Machine Learning in recent years there is also a new trend that combines traditional mathematical methods with compensation networks (to correct errors and improve accuracy [15]).

Some of the laser 3D triangulation solutions assume that the cameras have already been calibrated, i.e., that the camera intrinsic parameters are known before doing the calibration of the whole triangulation system. The camera calibration is a standard and well known procedure, except for those cameras with Scheimpflug. Thus, with these adapters we are able to focus the laser projection in a wider range, which is a desirable property for a 3D triangulation system. This improvement is specially noticeable when dealing with objects of a considerable size, in which the laser beam hits the surface of the object at different distances (depths) from the camera. Despite its benefits, it also increases complexity, specially when doing the calibration of the camera (additional tilt parameters need to be estimated [2,4,5,19]).

Compared to the existing solutions, this work describes a 3D triangulation system that uses cameras with Scheimpflug adapters and proposes a calibration procedure that requires a 3D calibration pattern that has been carefully designed for this application. Thanks to the use of this specific 3D calibration pattern, the calibration process is quick and simple, without requiring much intervention.

## 3. Methodology of the Calibration Method

Classical methods consider a laser triangulation system composed by the set of cameras $C=\{c_{1},c_{2},\ldots ,c_{n}\}$ and the set of lasers $L=\{l_{1},l_{2},\ldots ,l_{m}\}$, each of them defining a local coordinate system. Given a point $P_{j}\in R^{3}$ that belongs to the plane defined by the laser $l_{j}$, its position $P_{i}\in R^{3}$ in the coordinate system defined by camera $c_{i}$ can be obtained as:

(1) $$P_{i}=T_{ij}\left(P_{j}\right)$$

where $T_{ij}\in SE\left(3\right)$ is the rigid transform relating the laser $l_{j}$ with the camera $c_{i}$.

This point can be expressed in a common reference frame by means of another rigid transform $T_{wi}\in SE\left(3\right)$ that relates the camera $c_{i}$ to the world coordinate system:

(2) $$P_{w}=T_{wi}\left(P_{i}\right)=T_{wi}T_{ij}\left(P_{j}\right).$$

The main objective of the calibration is to find all the transforms $\hat{T}\subset \left\{T_{ij},T_{wi}\right\}$ where $T_{ij}\in \hat{T}$ if the laser $l_{j}$ is visible by the camera $c_{i}$. In order to calibrate the laser frames respect to the observing cameras, most methods use planar checkboards [12] or even 3D calibration boards [20], placed such that so that the laser lines cut across squares of the checkboard. The drawacks to this type of calibrations is that the intrinsic parameters of the cameras have to be estimated with high accuracy, especially in the case of Scheimpflug cameras.

Our method assumes that the images are undistorted (either computing Scheimpflug distortion maps as calculated in the next subsections or that the distortion can be considered negligible). Instead of computing the previous matrices, we use a calibration object that can be easily detected in the images of the cameras, the method being similar to [21]. Given that all the points $P_{j}$ reflected by a laser are contained in the same plane, whose frame is defined having the Z coordinate equal to zero, the calibration can be computed in $P^{2}$ as the homography relating points in the laser plane to their projections in the image plane, that works both for regular and Scheimpflug cameras.

(3) $$p_{i}=H_{ij}p_{j}$$

where $p_{j}\in P^{2}$ is the point $P_{j}$ expressed in the local reference system of the laser plane $l_{j}$, and $p_{i}\in P^{2}$ is the corresponding point in the camera plane $c_{i}$ in pixels. Note that the intrinsic parameters of the camera are implicitly included in $H_{ij}$ if $p_{i}$ is expressed in pixel coordinates, which makes the calibration simpler and less error-prone.

In the same way, if a laser is visible in two or more cameras they can also be related by another homography, having as a result the relationship between all the reference systems. In case that there are several lasers, the transformation between the local laser frames can be derived by using calibration patterns that have a shape that favours the estimation of this transformation (see Section 4).

These homographies are recovered using the Direct Linear Transform (DLT) algorithm [22] and the corresponding transforms in $\hat{T}$ are finally extracted using the decomposition described in [23].

### 3.1. Scheimpflug Model

In this section, different methods to identify and correct the camera Scheimpflug distortions are shown, as well as identification of the cases in which image distortion correction is irrelevant.

#### 3.1.1. Projection Model

As shown in Equation (3), the relationship between the laser and the image planes, even in the case of different measurement units (e.g., millimeters in the laser plane and pixel in the image plane) is a homography in the absence of the image distortion correction, which also works for the case of Scheimpflug cameras. The camera projection in the case of tilted cameras is given by the equation:

(4) $$s{\left(x_{t},y_{t},1\right)}^{T}=KH_{p}{\left(x_{cam},y_{cam},1\right)}^{T}$$

where ${\left(x_{t},y_{t},1\right)}^{T}$ corresponds to the homogeneous coordinates of a point in the tilted camera frame in pixels, ${\left(x_{cam},y_{cam},1\right)}^{T}$ corresponds to the homogeneous coordinates of the observed laser point in the camera frame in normalized image coordinates (projection of the observed point into a camera with focal distance equal to 1), *K* corresponds to the intrinsic pinhole camera projection with principal distance *c* equal to the distance between the optical center and the camera sensor. $H_{p}$ corresponds to the transformation of points between the untilted and the tilted image planes, whose origin is at a distance $d=m_{p}*c$ from the projection center and $m_{p}$ corresponds to the pupil magnification factor.

Note that in the case that the plane is no tilted, $H_{p}$ is a unit matrix, so that the above equation corresponds to the standard pinhole camera projection. The transformation $H_{p}$ of points in the camera frame to the Scheimpflug plane is given by the following homography [5]:

(5) $$H_{p}=\left[\begin{array}{ccc} r_{11}r_{33}-r_{13}r_{31} & r_{21}r_{33}-r_{23}r_{31} & 0\\ r_{12}r_{33}-r_{13}r_{32} & r_{22}r_{33}-r_{23}r_{33} & 0\\ r_{13}/d & r_{23}/d & r_{33} \end{array}\right]$$

where $r_{ij}$ is the i,j element of the matrix $R_{t}$ that defines the rotation part of the transformation between the tilted and untilted frames.

The model described by Equation (3) remains valid since it can be expressed as a composition of homographies:

(6) $$p_{i}=KH_{p}{C_{N}}_{ij}p_{j}=H_{ij}p_{j}$$

where ${C_{N}}_{ij}$ is the normalized camera matrix with focal unit length [22], i.e., ${C_{N}}_{ij}=\left(\begin{array}{ccc} I & | & 0 \end{array}\right)\left(\begin{array}{ccc} R_{ij} & | & T_{ij}\\ 0 & | & 1 \end{array}\right)=\left(\begin{array}{cc} R_{ij} & T_{ij} \end{array}\right)$, and $R_{ij}$, $T_{ij}$ are the rotation an translation part of the pose of the laser frame *j* relative to the camera frame *i*.

#### 3.1.2. Distortion Model

The radial distorsion model of Brown [24] computes radial and decentering distortions in the normalized plane (plane at a unit focal length). In case of untilted cameras, radial distortions are accurate enough to model it. For the Scheimpflug model presented here, the transformation of pixels from the tilted plane at distance *d* to the normalized untilted unit plane is made with the transformation:

(7) $$s{\left(x_{u},y_{u},1\right)}^{T}=H_{p}^{-1}K^{-1}{\left(x_{t},y_{t},1\right)}^{T}$$

with the matrix $H_{p}$ being the unit matrix, as the plane at distance *d* is untilted, and $\left(x_{u},y_{u},1\right)$ corresponds to the camera projection in the untilted plane in normalized coordinates. Thus, the radial distortion calculation is made the same way as in a regular pin-hole camera. In order to calculate the distortion map in the tilted plane (in pixels), first the coordinates of the distorted point ${\left(x_{d},y_{d}\right)}^{T}$ are calculated:

(8) $$s\left[\begin{array}{c} x_{d}\\ y_{d} \end{array}\right]=\left[\begin{array}{c} x_{u}\left(1+K1r^{2}+K2r^{4}+\ldots \right)\\ y_{u}\left(1+K1r^{2}+K2r^{4}+\ldots \right) \end{array}\right];r^{2}={x_{u}}^{2}+{y_{u}}^{2}$$

Then, the distorted point in the tilted image plane in pixels results in:

(9) $$s{\left(x_{dt},y_{dt},1\right)}^{T}=KH_{p}{\left(x_{d},y_{d},1\right)}^{T}$$

**Figure 2:**
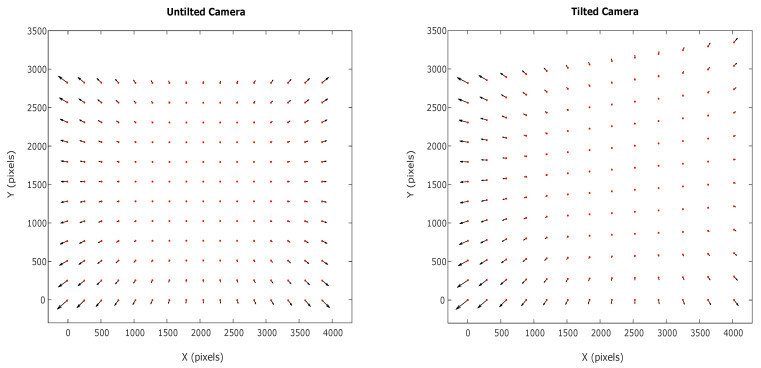
Distortion map errors amplified 10 times for an untilted and tilted camera by 25${}^{\circ }$ and magnification ratio $m_{p}=1$.

As an example, one of our cameras with 50 mm focal length is tilted 25${}^{\circ }$ with magnification ratio of 1. The untilted and tilted distortion map are shown in Figure 2. The Matlab code for the calculation for these maps has been included in Appendix A.

2

#### 3.1.3. Distortion Model Calculation

There are different methods to calculate the Scheimpflug distortion:

- Calculation of the distortion radial coefficients in case that a similar untilted camera is available. In this case the lens can be fitted into this camera and a standard camera calibration can identify the radial parameters. With this data, and the knowledge of the tilted Scheimpflug angle as well as the magnification factor $m_{p}$, the Scheimpflug distortion map can be calculated as in the previous section.
- Other alternatives consist of performing a Scheimpflug calibration as in [5], which has already been implemented in the Halcon-Mvtec software [25]. The identified (radial distortions and $H_{p}$ transformation matrix) allows one calculation of the Sheimplug distortion map.
- In cases where the tilted angle is small (Scheimpflug angles smaller than 6°), [4] showed that the decentering distortion parameters (also called tangential parameters) compensate the Scheimpflug angle effects. Thus, a pin-hole standard calibration with radial and decentering parameters can be used in order to calculate the Scheimpflug distortion map.

#### 3.1.4. Usage of the Scheimpflug Distortion

Many lenses with large focal lengths present almost no image distortion. In addition to this favorable scenario, in order to calculate the laser-camera homography without Scheimpflug distortion compensation, but with a given accuracy, one of the following procedures should be follow to ensure that the right conditions exist in order to discard distortion compensations:

- An initial approximated Scheimpflug distortion map can be estimated employing one of the previous methods. With the initial estimation of the laser-camera homography, the laser projections should be calculated for the pixels on the border of the image (the ones having largest distortions) with and without distortion. If the error obtained in the laser plane is smaller than the precision needed in the reconstruction, then the calculation of the accurate Scheimpflug map compensations can be avoided.
- Laser lines produced by the laser or other lines in the scene should be observed in the camera image. If the line fitting errors of the projected lines in pixels is similar to the errors in the estimation of the laser pick detector, Scheimpflug distortion maps can be avoided.

In the proposed proposed solution we have discarded the Scheimpflug distortion compensation because we have tested that we get the desired accuracy without applying it (see Section 6.1).

## 4. Implementation and Calibration for the Presented Setup

**Figure 3:**
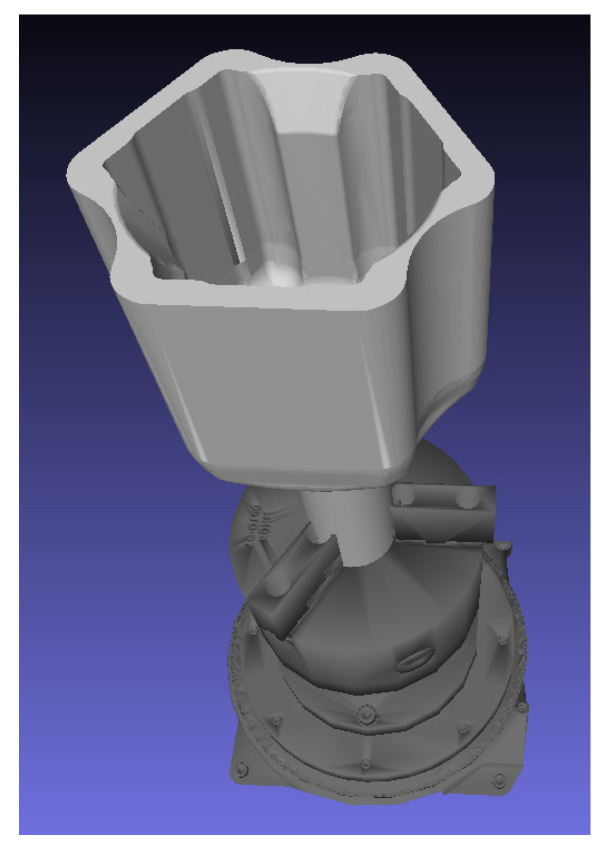
Example of revolution part that is handled with the proposed laser 3D triangulation system. The part (colored in light gray) is tied by the claws of the rotation stage (colored in dark gray).

Figure 1 represents our laser 3D triangulation system composed by two lasers and three cameras, plus a motorized rotation stage (model 8MRB240-152-59—Large Motorized Rotation Stage of Standa with an accuracy of 15’) that includes claws to fix the object. This setup has been specifically designed to capture revolution parts such as the one shown in Figure 3.

Two cameras and one laser have been placed at the top to obtain the reconstruction of the interior of the object (the second camera is intended to avoid occlusions). Similarly, the remaining camera and laser have been placed at the bottom to reconstruct the external surface of the object. Given that, the proposed system has 3 camera-laser pairs.

**Figure 4:**
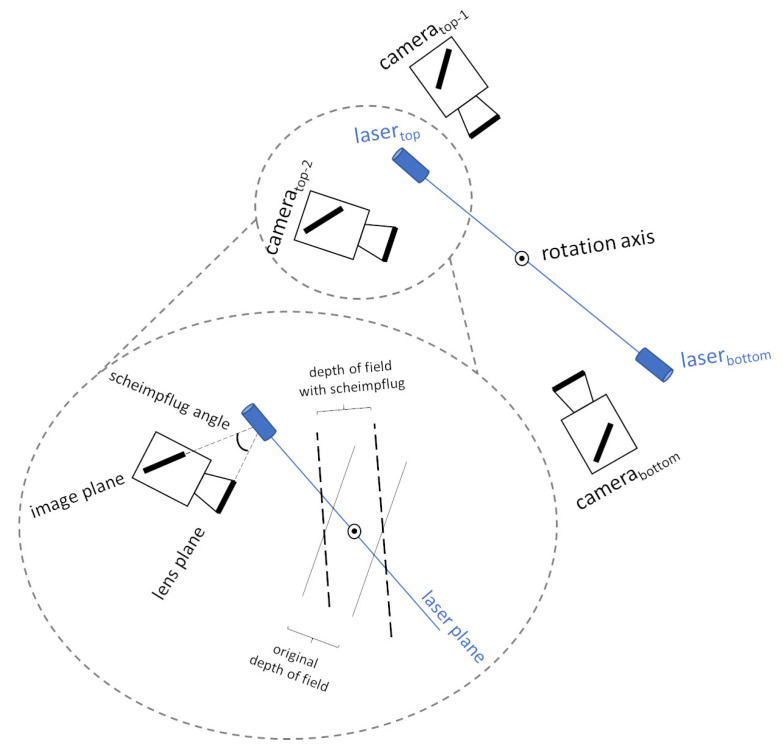
Arrangement of cameras and lasers in the proposed laser 3D triangulation system. The Scheimpflug principle is detailed for one of the camera-laser pairs.

Both lasers have been oriented in such a way that their projected lines are aligned with the rotation axis of the rotation stage, i.e., both laser projections and the rotation axis are coplanar. Furthermore, to ensure a focused image in the maximum area of the laser plane, each camera includes a Scheimpflug adapter to make its image plane and the corresponding laser plane as perpendicular as possible (Figure 4).

### 4.1. Modeling

Following the methodology depicted in Section 3, this setup is composed by three cameras $c_{1},c_{2},c_{3}$ and two lasers. However, as both lasers are coplanar, the setup can be simplified as a single laser configuration with $l_{1}$ in a first step, although an alignment refinement is presented in Section 4.4.5.

With this assumption, the system is completely described by Equation (6) as:

(10) $$\begin{array}{c} p_{c1}=H_{1}p_{l1}\\ p_{c2}=H_{2}p_{l1}\\ p_{c3}=H_{3}p_{l1} \end{array}$$

where $H_{1},H_{2},H_{3}$ are the homographies relating the laser plane with the three cameras.

To get a 3D reconstruction of an object, it is placed on the rotation stage and a complete rotation (360 degrees) is performed automatically (controlled by the motorized rotation stage). It is noteworthy that the piece is fixed with claws to center it in the stage, as well as to avoid unexpected displacements during the rotational motion.

For each rotation step (1 degree in our case), each camera-laser pair takes a capture, so that each camera-laser pair provides 360 profiles in total. Note that this movement is analytically equivalent to a setup with 360 laser planes, where each plane $l_{j}|1\leq j\leq 360$ is related to a world plane with a rotation $R_{j}$ around the axis of the stage.

Without loss of generality, if we assume that the axis of the stage is at the origin of the reference system, the reconstruction of a point $p_{w}$ captured by the camera *i* at the step *j* can be expressed as follows:

(11) $$\begin{array}{c} p_{w}=R_{j}H_{i}^{-1}p_{i} \end{array}$$

### 4.2. Calibration Pattern

**Figure 5:**
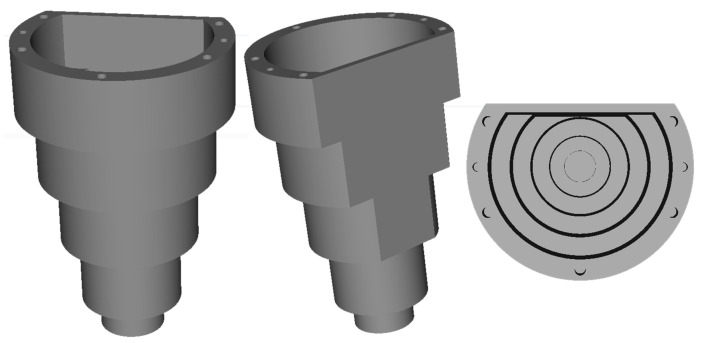
Calibration pattern. Front (**left**), back (**middle**) and top (**right**) views of the 3D model.

The calibration pattern that has been used is the one shown in Figure 5. It has a conical shape and it is formed by five coaxial cylinders of different diameters to which a cut has been applied to get two planar faces (inner and outer) with the same orientation. Its design has been inspired by the one used in [8], but a planar face has been added to be able to solve the degree of freedom associated with the turn about the axis of rotation (see below).

This design has been chosen for the following reasons:

- When a laser beam hits the pattern in the part of the cylinders, the beam describes a staircase shape and its intersection points can be detected and act as control points to get the calibration homographies (see Section 4.4.1 and Section 4.4.2). This staircase shape appears on both sides of the pattern, on the outer face as well as on the inner face. The outer face is visible by the camera at the bottom while the inner face is visible by the two cameras at the top.
- After having an initial solution of the calibration, a 3D reconstruction of the calibration pattern can be performed. In this 3D reconstruction, the two planar faces can be detected automatically and used to refine the alignment between the top and bottom camera-laser pairs along the axis of revolution (see Section 4.4.5).

### 4.3. Laser Capture

The accuracy of a 3D reconstruction using laser linear illumination is significantly determined by the accuracy of the line segmentation in the image. Since the pattern of image intensity in the normal direction to the line has a Gaussian profile, finding the center of the line in the image corresponds to detect the point of maximum intensity in the normal direction, i.e., the laser peak. This peak can be detected by different algorithms using the intensity distribution along a column of the sensor image, e.g., finding the position of maximum intensity, finding thresholding points or finding the center of gravity [26,27,28]. In the proposed sensor, a Savitzky-Golay [29] finite impulse response (FIR) differential filter is applied to the image intensity profile of the laser line computing the zero-crossings with sub-pixel accuracy [30,31,32].

### 4.4. Method

**Figure 6:**
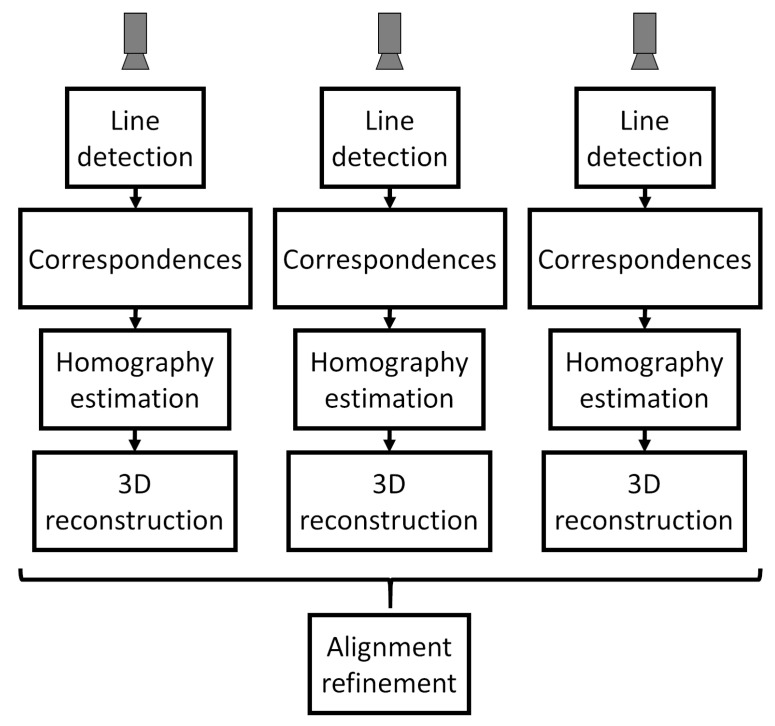
Calibration pipeline.

In order to calibrate the system, the calibration pattern is placed on the rotation stage and rotated 360 degrees while each camera-laser pair takes a capture per degree. After capturing this set of profiles, the steps shown in Figure 6 are executed for camera-laser pair to automatically extract the corresponding homographies.

**Figure 7:**
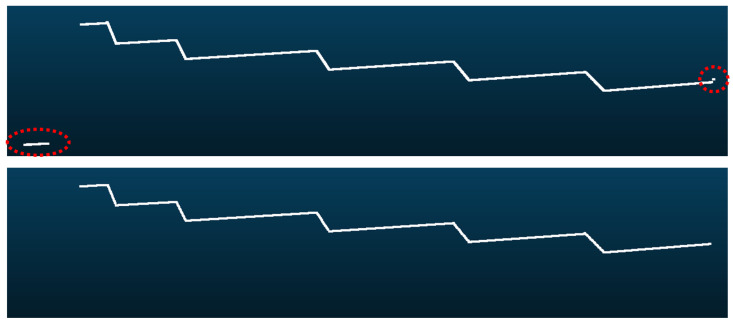
Example of captured laser profile for the calibration pattern. Raw data with noise highlighted in red (**top**), and the result after removing noise and detect lines (**bottom**).

The shape of the calibration pattern (Section 4.2) generates images that have a staircase shape as shown in Figure 7. For each image generated by each camera-laser pair a line detection algorithm is applied to extract predominant lines (Section 4.4.1). The intersection points of these lines are matched to their reference (nominal) counterparts that are specified in a global coordinate system (Section 4.4.2). Using these correspondences, for each camera-laser pair the homography that transforms points from the image plane to the laser plane can be estimated (Section 4.4.3). Additionally, the 3D reconstruction for each camera-laser pair is obtained by applying the corresponding rotation angle (given by the encoder of the rotation stage) using Equation (11) (Section 4.4.4). Therefore, each partial 3D reconstruction is accumulated to build the final 3D reconstruction. In the last refinement step these partial 3D reconstructions are aligned with each other using the planar reference faces of the calibration pattern. This faces are automatically detected so that the final homographies make them share the same normal (Section 4.4.5). An overview of the whole pipeline is shown in Figure 6.

#### 4.4.1. Line Detection

Figure 7 shows an example of a captured laser profile of the calibration pattern at each rotation step. The raw captured profile contains some noise (highlighted in red in the top of Figure 7), especially at the extremes, since the laser beam can hit other objects apart from the calibration pattern. As this noise usually appears at the extremes and is separated from the main part, to remove it a point clustering is done based on the euclidean distance, and the biggest cluster is only retained.

**Figure 8:**
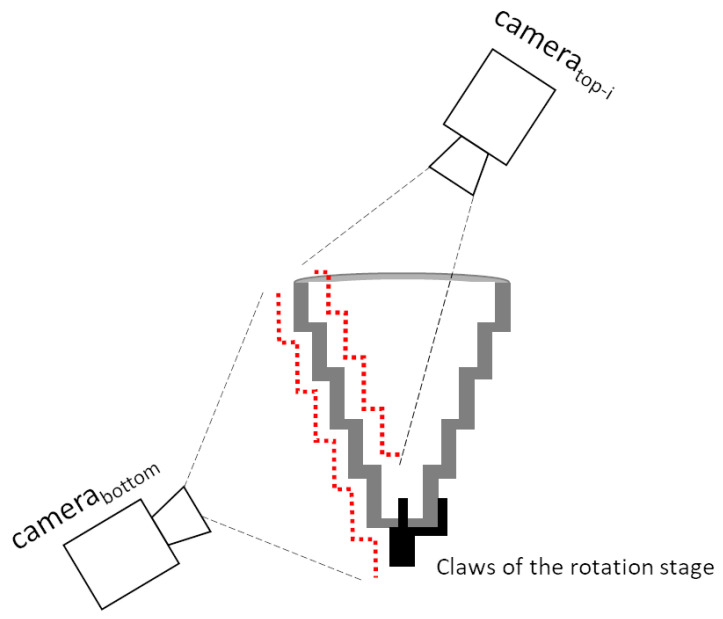
Maximum number of lines of the calibration pattern that are visible by each camera-laser pair in each profile (highlighted in red). The body of the claws of the rotation stage increases the number of lines for the bottom camera-laser pair. The bottom part of the inner side of the calibration pattern is not visible by the top cameras.

With the cleaned profile, *N* predominant lines are searched using RANSAC [33], which is a hypothesis-verification technique. We execute RANSAC *N* times or until no points are available, and for each execution the predominant line is obtained and its inlier points (those points whose distance to the line is less than a predefined threshold) are removed from the profile for the next execution. *N* represents the maximum number of lines to be detected for each profile and is a predefined parameter that depends on the shape of calibration pattern and its visibility from the camera. Taking into account visibility limitations, in our configuration, we have set *N* as 11 for the bottom camera-laser pair and 9 for the top camera-laser pairs (see Figure 8 to substantiate how these numbers are defined). It should be emphasized that less than *N* lines will be detected in the planar side of the calibration pattern because the stop criterion will be given by the absence of more available points to form line hypothesis.

To avoid undesired line detections, such as the line that crosses the entire profile and which would have a large number of positive votes in RANSAC, we have included the following heuristics to the original RANSAC algorithm.

- *Length restrictions:* Instead of considering 2 random points to form a line hypothesis, we only consider those pairs of points whose distance is between a predefined range. Thus, we discard hypotheses that are formed by too close points (which offers unstable estimation of the line direction) as well as those formed by too distant points (to reduce the appearance of hypotheses with considerable length). When assigning positive votes to a line hypothesis, we also discard those points that are too distant from the 2 original points that form the line hypothesis.
- *Direction restrictions:* Given the staircase shape of our profile, the new line to be detected in the current RANSAC execution has to be almost 90 degrees from the line detected in the previous execution, i.e, we use the normal of the previous detected line as an initial estimation of the direction for the current line. We estimate the normal for each point using the *k* closest points at the beginning, so that line hypothesis are only formed by point pairs that have similar normal. Similarly, when assigning positive votes to a line hypothesis, we discard those points whose normal is not similar to the normal of the line hypothesis.

Additionally, the line estimated after each RANSAC execution need to pass a *fitting quality* control to be considered as valid. Within the inlier point set of the line, the two most extreme points are taken to form the longest segment, and its oriented bounding box is calculated and used to retain those points from the whole profile that are inside it, i.e., to get a potential inlier point set. Thus, the ratio *number_of_inlier_points/number_of_potential_inlier_ points* represents the fitting quality and must be higher than a predefined value to register the current line as a good one. This test is mainly focused on discarding lines with a spatially non-uniform and dispersed set of inliers (generally produced by several groups of independent points), and which is usually associated with a bad estimate.

To speed up all the calculations (including the initial point clustering), we work on a subsampled profile (we apply a non-maximum suppression), and once we detect the lines, we refine them using the original profile points to improve the accuracy of line estimation.

#### 4.4.2. Correspondences

After the previous step, we have several line estimations for each profile, and each line can be represented by the longest segment formed by the the two most extreme points of the inlier set. Thus, for each profile we sort its segments using the coordinates of these points (those closest to the origin first), and then, compute the intersections between consecutive segments. To avoid bad configurations, we only retain those intersection points that are close to one of the extreme points of both segments. Moreover, from the whole set of 360 profiles, we initially select those for which the number of intersection points is equal to the number of nominal intersections.

**Figure 9:**
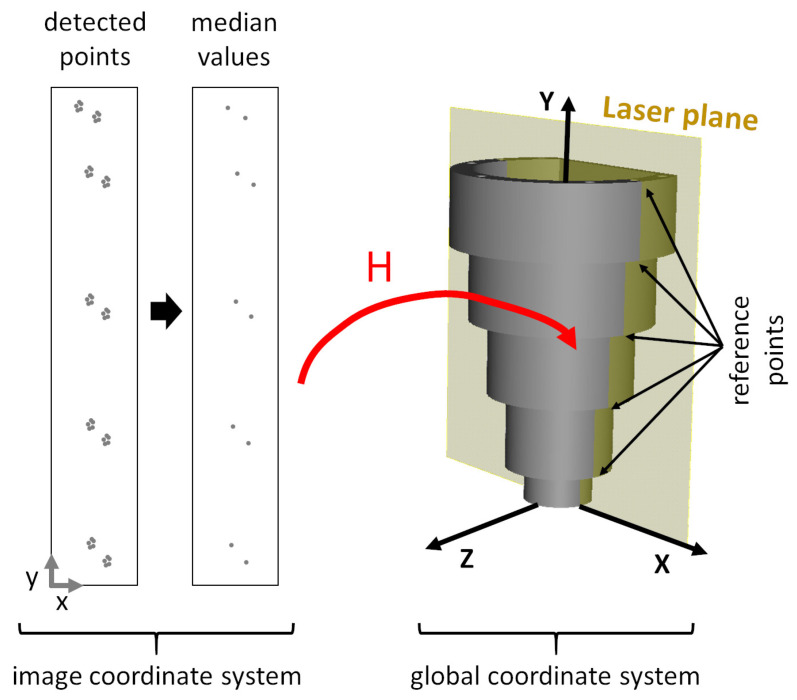
Conceptual representation of the homography estimation. Intersection points of the whole profile set are simplified using median values and used as correspondences of the reference intersection points. The computed homography transforms points from the image plane (in image coordinates) to the laser plane (in global coordinates).

These selected profiles provide an unambiguous correspondence between detected and nominal intersection points, which are the control points used to calculate the homograpy between the camera and the laser plane. The nominal intersection points have been obtained by measuring the calibration pattern using a Coordinate Measuring Machine (CMM). In our case, we have used the Mitutoyo Crysta-Apex S 9106 CMM model to do this measurement, which offers a high accuracy (∼0.002 mm). As the cylinders of the pattern are symmetrical about the central axis of the pattern (which coincides with the axis of rotation of the system), the CMM has measured the intersection points along a plane that cuts the pattern and passes through the central axis, which is coincident with the laser plane (see Figure 9 for a graphical representation).

Many times the set of selected profiles are concentrated in one small area of the calibration pattern, so the posterior results can be overfit to this area. Furthermore, since the calibration pattern has manufacturing tolerances, the results may vary depending on the area used. To avoid this problem, we make a second pass to try to include some intersection points from unselected profiles and get correspondences that are distributed in a more uniform way throughout the entire surface of the calibration pattern. Using the intersection points of the selected profiles, we compute the median for each of the N−1 intersection points, and then, for each unselected profile we check if its intersection points are close to these median values. In case of finding at least 2 valid intersection points, the unselected profile is considered to be partially good and its validated intersection points are included to the set of correspondences.

#### 4.4.3. Homography Estimation

The previous step provides a set of correspondences between the intersection points detected in the image and the reference intersection points that are in global coordinates and rest on the laser plane. More precisely, for each of the N−1 different reference intersection points we have several correspondences. Under our experience, instead of using a RANSAC style algorithm with all correspondence samples, we have had more stable results making the median of the detected image correspondences for each of the N−1 different reference points, and then, computing the homography (in a least squares sense) using the resultant N−1 correspondences (Figure 9). The median values are used to compensate possible manufacturing tolerances (eccentricities, etc.) of the calibration pattern.

The homography that is estimated transforms points from the image plane to the laser plane (or viceversa if its inverse is used, since a homography is a 3 × 3 invertible matrix). For clarification purposes, we assume that the laser plane coincides with the XY plane of the global coordinate system, i.e., Z=0. Given that, and following the notation of Equation (11), the homography transformation chain can be expressed as

(12) $$\begin{array}{cc} p_{w} & ={\left(X,Y,W\right)}^{T}=H_{i}^{-1}{\left(x,y,1\right)}^{T}=H_{i}^{-1}{\left(p_{i},1\right)}^{T}\\ P_{w} & ={\left(X/W,Y/W,0\right)}^{T} \end{array}$$

where $p_{w}$ are the XY homogeneous coordinates of the laser points, $P_{w}$ are the 3D coordinates of the laser point in the global coordinate system.

#### 4.4.4. 3D Reconstruction

According to Equation (12) points of each of the 360 captured profiles can be transformed to the global coordinate system. However, this transformation moves all points to the laser plane, i.e., all the profiles are accumulated in the same plane. To reconstruct properly each profile we have to apply the corresponding rotation to each profile. When a profile is captured, we know at which rotation step has been captured, since this information is provided by the encoder of the rotation stage. Thus, the points of the profile *k*, which has been captured at the rotation step *j* (which equals to *j* degrees, as 360 profiles are captured in 360 degrees, i.e., rotation step is 1 degree), must be transformed as follows

(13) $${\left(X^{\prime },Y^{\prime },Z^{\prime }\right)}^{T}=\left[\begin{array}{ccc} cos(j) & 0 & sin(j)\\ 0 & 1 & 0\\ -sin(j) & 0 & cos(j) \end{array}\right]P_{w}^{k}=R_{j}P_{w}^{k}$$

where $P_{w}^{k}$ represents the points of the profile *k* after applying Equation (12), $R_{j}$ is a rotation matrix of *j* degrees about the axis of rotation of the system (which coincides with the Y axis of the global system), and  ${\left(X^{\prime },Y^{\prime },Z^{\prime }\right)}^{T}$ are the resultant global coordinates of the point and which are used to build the 3D reconstruction of the scanned object (the calibration pattern).

#### 4.4.5. Alignment Refinement

All the processing steps described in the previous Section 4.4.1, Section 4.4.2, Section 4.4.3 and Section 4.4.4 are executed by each camera-laser pair independently. As a result, we obtain several partial 3D reconstructions of the calibration pattern, one for each camera-laser pair. These partial 3D reconstructions are correctly aligned in translation and in the rotations of the *X* and *Z* axes, but the alignment in the rotation of the *Y* axis (the main axis of the rotation stage) is not guaranteed, since each camera-laser pair has a different origin of the rotation (different starting point) through the *Y* axis when applying Equation (13). If this rotation ambiguity is not solved, the partial reconstructions are not well aligned on the *Y* axis, and therefore, if these partial reconstructions were joined, a bad global reconstruction would be seen (there would be an offset between them). This misalignment effect would be appreciated especially in those objects that are not completely symmetrical along the *Y* axis.

To ensure a correct alignment in the rotation of the *Y* axis we use the planar faces of the calibration pattern (Figure 5). In each partial 3D reconstruction we can search for the predominant plane and use its normal to align them all together, i.e., find a rotation delta in *Y* axis ($\Delta_{i}$) for each camera-laser pair (*i*) to force that all the plane normals match and point in the same direction. The final global coordinates ${\left(X^{\prime \prime },Y^{\prime \prime },Z^{\prime \prime }\right)}^{T}$ that are used to build the 3D reconstruction of the scanned object (the calibration pattern) are obtained after applying this rotation delta to the Equation (13).

(14) $${\left(X^{\prime \prime },Y^{\prime \prime },Z^{\prime \prime }\right)}^{T}=\left[\begin{array}{ccc} cos(\Delta_{i}) & 0 & sin(\Delta_{i})\\ 0 & 1 & 0\\ -sin(\Delta_{i}) & 0 & cos(\Delta_{i}) \end{array}\right]{\left(X^{\prime },Y^{\prime },Z^{\prime }\right)}^{T}$$

Given that, it is noteworthy that the calibration of each camera-laser pair (*i*) will be given by its own homography ($H_{i}$) and rotation delta ($\Delta_{i}$) as shown in Equation (12)–(14). Likewise, the final 3D reconstruction will be given by merging all partial 3D reconstructions of all camera-laser pairs.

The extraction of the predominant plane for each partial 3D reconstruction can be speed up using the grouping of profiles done in Section 4.4.2. The profiles that were discarded as correspondences for not having multiple intersection points between lines, are precisely those that will belong to the part of the calibration pattern where the plane is located. Therefore, we can identify these profiles and only use their 3D reconstruction to find the predominant plane.

## 5. Reconstruction of Revolving Objects

Previous section describes the calibration process of the proposed 3D laser triangulation system. Thus, once the system has been calibrated, it can be used to build the 3D reconstruction of revolving objects, such as the one shown in Figure 3.

The procedure to scan and reconstruct an object is straightforward. The user has to place the desired object in the rotation stage and ties it. Then, the user pushes a button and the system automatically applies a complete rotation (360 degrees) while capturing profiles with the different camera-laser pairs. Afterwards, the system uses the calibration data ($H_{k}$ and $\Delta_{k}$) of each camera-laser pair (*k*) to apply Equations (12)–(14) to the corresponding profiles and to obtain the 3D reconstruction of the object.

## 6. Experiments and Results

### 6.1. Scheimpflug Distortion

In our experiment an approximated Scheimpflug distortion map for the used cameras (C5-4090-GigE cameras from Automation Technology Gmbh and Zeiss Planar T* 50 mm lenses from Nikon) and the estimated homography were calculated resulting in maximum errors of 2 microns. These errors comes from the fact that a similar camera but without Scheimplug angle, i.e., perpendicular camera, has been calibrated with the same lens used in the Scheimplug camera. A very precise dot calibration pattern with precision of about 2 microns has been used to calibrate the perpendicular camera, noticing practically no radial distortion, with a re-projection error that comes from the errors of the calibration plate. The Scheimplug distortion map, calculated with the matlab code of Appendix A, shows distortions of a similar magnitude that the distortions of untilted camera.

Real calibration of the laser-camera homography was made by fitting projecting the laser over a calibrated revolution cone, assuming that the distortion map can be avoided. The errors obtained in the homography estimation were smaller that 7 microns. The advantage of this method resides on the fact that difficult and error-prone Scheimpflug calibration procedures can be avoided, which can at the end be more costly and less precise than this simpler method.

### 6.2. Calibration Repeatability and Accuracy

In order to demonstrate the repeatability of the proposed calibration method, we have performed the calibration 10 times. Moreover, to introduce variability in the data, the pattern has been placed in a different initial position for each calibration, providing a different set of profiles as input. At each calibration execution we have calculated the calibration data and the 3D reconstruction of two different objects: (1) the calibration pattern, and (2) an object that is similar in shape to the calibration pattern, but with a small change in the size (different height and different diameter lengths). The idea of introducing this second object is to see results without the possible effects of overfitting due to the usage of the same object for both calibration and test.

**Figure 10:**
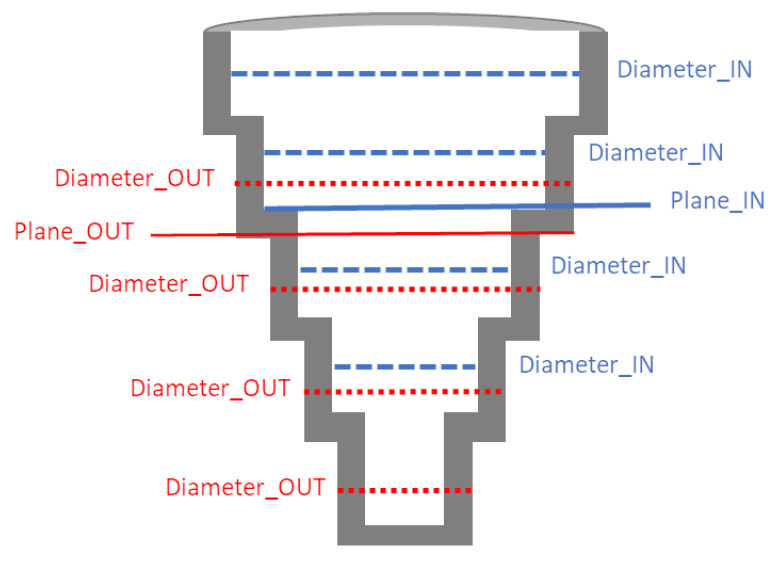
Measured cylinders and planes for each calibration execution during the repeatability experiments (see text for details). Inner cylinders and plane are highlighted in blue, while outer cylinders and plane are in red color.

We have used these 3D reconstructions to perform some measurements and provide a comparison between them. More precisely, we have measured the diameter of several cylinders. Furthermore, the height of a plane at different locations has been estimated for the calibration pattern as well (see Figure 10 for a graphical representation of measured cylinders and planes). In all cases we have used points of the inner and outer part of the 3D model to estimate cylinders and planes of the outer and inner parts respectively.

Table 1 and Table 2 show the error (expressed in millimeters) of these measurements respect to the nominal values, which have been obtained using a CMM (Mitutoyo Crysta-Apex S 9106, which offers a high accuracy, ∼0.002 mm).

To provide a robust central tendency, truncated mean of the results of the 10 calibrations has been calculated, i.e, extreme values (best and worst) have been discarded during the mean computation. Moreover, the standard deviation of the error of these 10 calibrations is shown for each measurement. These standard deviation values are low, which indicates that the calibration algorithm offers a good repeatability.

To obtain each measurement (related to cylinder or plane), points of the 3D reconstruction that are close to the corresponding nominal value have been sampled, and then, a specified fitting algorithm has been applied. Thus, it is noteworthy that the errors that are shown in Table 1 and Table 2 accumulate the error of: (i) the laser (sensor accuracy), (ii) the centering of the object on the axis of rotation, (iii) the proposed calibration method and (iv) the fitting algorithms. In fact, the effect of the error in the centering of the object, which mechanically depends on the claws that tie the object at its bottom, is noticeable by observing the errors, since in the upper and outer areas of the object there is more error than in the lower and bottom areas respectively (a slight pitching effect).

As an supplementary measure of accuracy, we have calculated the distance between the 3D reconstructed model and its corresponding original 3D model for the object with similar shape. We have compared each of the previous 10 3D reconstructions of this object against its original 3D model. The mean and standard deviation of the errors of these 10 comparisons has turned out to be 0.151 mm and 0.015 mm respectively. As stated before, these errors values accumulate the error of several sources (the laser, the centering of the object, the quality of the calibration and the fitting algorithm).

Taking into account all the experiments presented above, we can point out that the system offers an accuracy of tenths of a millimeter with a variability of hundredths of a millimeter.

#### Real Case

As an additional evidence of the validation of the proposed system, we have made experiments with an industrial part (Figure 3) that is being used in a real factory. We have used it as a reference to validate the accuracy of the calibration. Being a result of an industrial manufacturing process, this part undergoes a series of measurements (designed and defined by expert metrologists) at some key points to ensure that the tolerances are met. These measurements are usually performed using a CMM, which offers high precision results. Thus, we have considered several of these measurements and we have calculated them using both the CMM and a metrology software which we have provided as input the scanned and reconstructed 3d model of the part using the laser 3d triangulation system described above (Section 4).

Comparing the results of both alternatives, we observed that the errors are less than 0.1 mm, which indeed, is accurate enough to be used for this real application. The details of the repeatability and reproducibility (R&R) experiments that led to this value can be found in [8].

### 6.3. Discussion

The proposed calibration methodology requires the use of a calibration pattern, whose main design features have been defined in Section 4.2. Getting a similar pattern can be a laborious task, but it only needs to be done once. In return, the proposed calibration process itself is fast (it takes less than 1 minute), simple, and requires minimal user intervention. Summing up, the user places the calibration pattern on the rotation stage and ties it, and then, the system automatically applies a complete rotation (360 degrees) while capturing profiles with the different camera-laser pairs. Afterwards, the system process automatically each profile to extract the lines, the correspondences against the reference points and estimate the calibration data. It should be noted that the automatic processing of each profile has proven robust to the noise that can appear during the capture of profiles.

The experiments have been carried out using two different surface materials. The calibration object is made of machined steel finished with a dark coating which has a certain specular component, while the validation object is made of plastic resin that has a greater diffuse component. In both cases we have observed an adequate behavior of the optical system, requiring only to adjust the exposure time of the cameras. Additionally it has also been tested with forged steel objects. It is noteworthy that very specular materials, such as glass or machined metals, are not suitable for the proposed system.

Considering the intended use we wanted to give to the proposed laser 3d triangulation system (emphasize simplicity and automaticity of use within some bounds of accuracy), we have discarded the use of the Scheimpflug distortion. The impact on accuracy (microns according to our experiments of Section 6.1) is not significant, and it simplifies greatly the calibration process, allowing us to offer an almost automatic solution.

Apart from being synchronized with a rotation stage, the proposed calibration method requires knowledge of the coordinates of the reference control points (Section 4.4.2) to perform automatically all the processing. Given that, our calibration method could be adapted to work with other calibration patterns just by providing the new coordinates of the reference control points. The requirement for these new calibration patterns is that they must generate a staircase shape in the profiles when the laser hits their surface (see Figure 7).

## 7. Conclusions

This paper describes a laser 3d triangulation system oriented to revolving pieces. It combines 3 cameras and 2 lasers to capture and reconstruct most of the surface of the objects that want to be scanned. Moreover, it includes Scheimpflug adapters to offer more flexibility in the setup of the cameras and lasers, i.e., to maximise the focus of the depth of field of the camera on the laser plane despite the orientation of both devices. Aside from the flexibility of the setup, another advantage of the proposed system is that it is easy to use, offering a high degree of automation of the entire process. The object to be reconstructed is automatically rotated using a rotation stage, and the 3 camera-laser pairs are synchronized in such a way that in each rotation step profiles are captured and processed automatically. The entire scan and reconstruct process takes less than 1 minute for each object, so it is another point in favor of the proposed 3d triangulation system as well.

Likewise, a fast and automatic calibration method is proposed to fine-tune the system. This calibration method uses a calibration pattern that has been designed to generate a staircase shape when the laser planes intersect with its surface. Indeed, this shape is what allows to perform an automatic processing of profiles in search of lines, their intersections, generate correspondences against the reference control points and to estimate the calibration data. For the calibration process, the calibration pattern is treated as an object in the sense that the user places the pattern on the rotation stage and it is rotated automatically to capture the profiles. Thus, the proposed calibration method is automatic and takes less than 1 minute. In order to achieve this high degree of automaticity and speed in calibration, the effect of Scheimpflug distortion has not been considered. Nonetheless, this has little impact on accuracy when using 50mmm lenses (microns, according to our experiments). Finally, in cases where the Scheimpflug distortion cannot be avoided, simple procedures for its calculation and the conditions were it can be avoided have been also presented.

The presented experiments validate the accuracy of the proposed laser 3d triangulation system when compared against the measurements made with a CMM. We have reported an accuracy of few tenths of a millimeter, which could be enough for several applications. In fact, as an evidence of its potential use, this system is already being used in a real factory to control the quality of the manufacturing process of an industrial part.

## Figures and Tables

**Table 1:** Mean and standard deviation error (in mm) of some primitives (cylinders and planes) of the calibration pattern respect to nominal values using different calibrations.

Calibration Pattern		Error	
**Measurement**	**Nominal**	**Mean**	**Stdev**
Diameter_OUT	44	0.064	0.009
Diameter_OUT	71	0.115	0.005
Diameter_OUT	97.01	0.017	0.005
Diameter_OUT	123	0.059	0.016
Diameter_IN	50.94	0.031	0.008
Diameter_IN	76.95	0.067	0.041
Diameter_IN	102.95	0.084	0.087
Diameter_IN	129	0.18	0.047
Plane_OUT	121.96	0.194	0.014
Plane_IN	132	0.061	0.012

**Table 2:** Mean and standard deviation error (in mm) of some primitives (cylinders) of an object, which is similar in shape to the calibration pattern, respect to nominal values using different calibrations.

Object with Similar Shape		Error	
**Measurement**	**Nominal**	**Mean**	**Stdev**
Diameter_OUT	72.044	0.126	0.011
Diameter_OUT	98.062	0.244	0.014
Diameter_OUT	124.075	0.268	0.031
Diameter_OUT	150.091	0.053	0.06
Diameter_IN	46.948	0.068	0.016
Diameter_IN	71.99	0.055	0.04
Diameter_IN	96.96	0.1	0.094
Diameter_IN	121.992	0.107	0.142
